# Supplementary material for: Cascade switching current detectors based on arrays of Josephson junctions
Source: Nat Commun. 2025 Aug 25;16:7927. doi: 10.1038/s41467-025-63360-y (PMC12379658; doi:10.1038/s41467-025-63360-y)
Supplement: Supplementary file 1 — Supplementary Information [file 41467_2025_63360_MOESM1_ESM.pdf]

# Supplementary information: Cascade switching current detectors based on arrays of Josephson junctions

Roger Cattaneo,[1] Artemii E. Efimov,[1,2] Kirill I. Shiianov,[1] Oliver Kieler,[3] and Vladimir M. Krasnov[1,*]

[1]*Department of Physics, Stockholm University, AlbaNova University Center, SE-10691 Stockholm, Sweden.*
[2]*Department of Physics, University of Basel, 4056 Basel, Switzerland*
[3]*Physikalisch-Technische Bundesanstalt, 38116 Braunschweig, Germany*

## SI. OPERATION OF A SWITCHING CURRENT DETECTOR

Detection of electromagnetic waves (EMW) by an overdamped current-biased JJ (CBJJ) has been well studied (for an overview see e.g. Ch. 11.5 in Ref. [1]). Unlike the SCD, CBJJ detector is biased at a constant current slightly above $I_c$. The EMW induces a small high-frequency current in the JJ, which suppresses the critical current and leads to the increase of voltage, $\Delta V = R_d \Delta I_c$, where $R_d = dV/dI(I)$ is the differential resistance of the current-voltage ($I$-$V$) characteristics at the bias point. The sensitivity $S$(V/W) of CBJJ is proportional to $R_d$, which is large at $I \simeq I_c$.

SCD based on an underdamped JJ is different from CBJJ detector. Underdamped JJs switch abruptly from the superconducting to the resistive state. For tunnel JJs the voltage jumps from zero to a large sum-gap value, $V_g = 2\Delta/e$, where $\Delta$ is the superconducting energy gap. In this case $R_d = \infty$ and the device operation can be described only statistically in terms of switching probabilities. A detailed analysis of SCD based on a single underdamped JJ can be found in Ref. [2].

Fig. S1 illustrates the operation principle of SCD based on a single underdamped JJ. Fig. S1 (a) represents the $I$-$V$ characteristics of a current-biased underdamped JJ. It exhibits a hysteresis: for increasing current it switches from the superconducting to the resistive state at a switching current $I_s \lesssim I_{c0}$, where $I_{c0}$ is the fluctuation-free critical current. Upon decreasing current it returns back to the superconducting state at a smaller retrapping current $I_r$. The SCD is biased by an ac-current at a low bias frequency $f_b$ (23 Hz for the data presented in the manuscript) with an amplitude $I_b \lesssim I_s$, as shown by the olive line in Fig. S1 (b). The impacting EMW induces an additional high-frequency current superimposed on the bias current, as sketched by the blue and red lines in Fig. S1 (b) for the cases of low and high impacting power, respectively. This causes both the enhanced probability of switching at lower current and a longer excursion in the resistive state. Fig. S1 (c) illustrates voltage responses of the JJ without radiation (olive line, $V = 0$, no switching) and with radiation at different EMW power (blue and red).

Figs. S1 (d-h) illustrate the stochastic nature of SCD response in a longer time frame. Here panel (d) shows time-dependence of the bias current. Panels (e-h) represent voltage responses upon increasing the radiation power. Without radiation, panel (g), there are few spontaneous switchings, which occur at the maxima of bias current. With increasing power, (f), the switching probability increases and more switching are observed. At a certain power, (g), switchings occur at every bias cycle. Further increase of power, (h), does not increase the low-frequency component of voltage, leading to saturation of the detector.

## SII. THERMAL ACTIVATION IN A JOSEPHSON JUNCTION

Dynamics of a JJ is equivalent to motion of a particle in a tilted washboard potential [2], $U(\varphi) = E_{J0}[1 - \cos\varphi - i\varphi]$, as sketched in Fig. S2 (a). Here $\varphi$ is the Josephson phase difference, $E_{J0} = (\Phi_0/2\pi)I_{c0}$ is the Josephson energy and $i = I/I_{c0}$. The washboard has bias-dependent parameters: the barrier height, $\Delta U = 2E_{J0}[(1-i^2)^{1/2} - i\arccos(i)]$, the eigenfrequency $\omega_p \simeq \omega_{p0}(1-i^2)^{1/4}$, and the quality factor, $Q = \omega_p R_{QP} C$. Here

$$\omega_{p0} = \sqrt{\frac{2\pi}{\Phi_0}\frac{I_{c0}}{C}}$$

is the zero-bias plasma frequency, $I_{c0}$ is the fluctuation-free critical current, $\Phi_0$ is the flux quantum, $C$ is capacitance and $R_{QP}$ is the low-bias (subgap) quasiparticle (QP) resistance, which for tunnel JJs is much larger than the high-bias normal resistance, $R_n$. We consider underdamped JJs with a quality factor,

$$Q_0 = \omega_{p0} R_{QP} C \gg 1.$$

Fluctuations cause premature escape out of the well at a switching current $I_s < I_{c0}$. The escape rate in the absence of radiation can be written as [2, 3],

$$\Gamma_0(I) = a(I)\frac{\omega_p(I)}{2\pi}\exp\left[-\frac{\Delta U(I)}{k_b T}\right]. \quad \text{(S1)}$$

The probability density for switching in the bias interval $I_b < I < I_b + dI$ is

$$g(I) = \frac{\Gamma(I)}{dI/dt}\left[1 - G(I)\right], \quad \text{(S2)}$$

* Vladimir.Krasnov@fysik.su.se

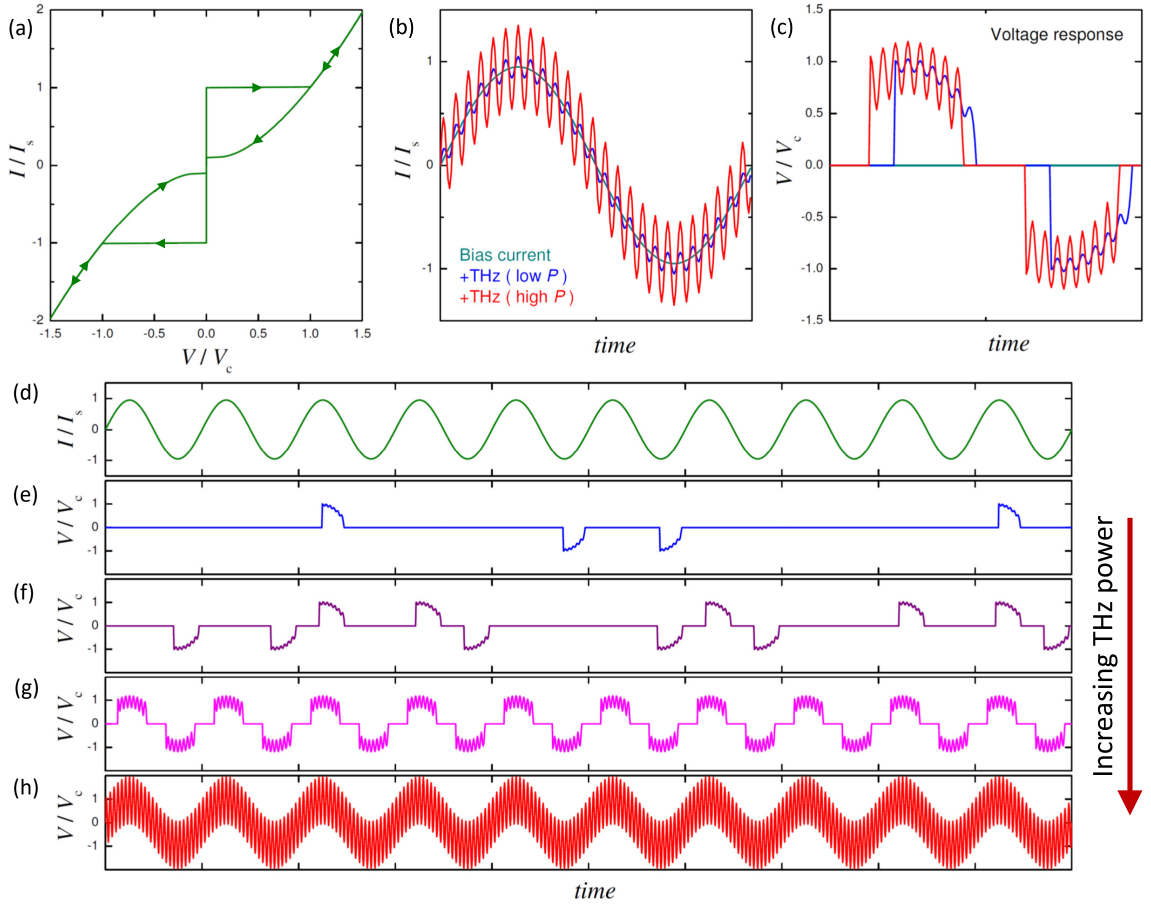


FIG. S1. **Operation principle of SCD, based on a single underdamped Josephson junction.** (a) The $I$-$V$ characteristics. (b) The olive line represents a sinusoidal in time bias current with the amplitude slightly below $I_s$. Blue and red lines represent time dependent currents through the JJ with superimposed high-frequency current induced by the impacting EMW. (c) Time dependence of the junction voltage for current profiles from (b). (d-h) Statistics of switching at longer times. (d) Time dependence of the bias current. (e-h) Time dependencies of junction voltages for increasing radiation power.

where $dI/dt$ is the bias ramp rate and

$$G(I) = \int_0^I g(I)dI \tag{S3}$$

is the total probability of switching upon ramping up to current $I$. Eqs. (S2) and (S3) form a recurrent equation, which can be easily solved numerically.

The navy lines in Figs. S2 (b) and (c) represent calculated bias current dependencies of (b) the probability density and (c) the switching probability for an underdamped JJ with $Q_0 = 100$, $I_{c0} = 50\ \mu$A at $T = 1$ K (from Ref. [2] ). They correspond to the experimentally measurable switching current histograms and time-average voltage, correspondingly.

The most important quantity for SCD operation is the width of switching histograms. In Ref. [4] it was shown that for SQUIDs, FWHM is proportional to $T^{2/3}$, which also holds for single JJ with $Q_0 \gg 1$,

$$\delta I_{s1} = a I_{c0} \left(\frac{k_B T}{2E_{J0}}\right)^{2/3}. \tag{S4}$$

According to our simulations, the prefactor, $a \sim 1$, slightly depends on $E_{J0}/kB_T$ and varies from $a = 0.57$ for $E_{J0}/k_BT > 100$ to $a \simeq 1$ for $E_{J0}/k_BT \sim 10$.

## SIII. SCD SENSITIVITY

Shaking of a wash-board potential by the EMW-induced current, Fig. S2 (a), leads to a premature switching of a JJ in the resistive state. Red and blue lines in Figs. S2 (b) and (c) represent calculated bias current dependencies of the switching histograms (b) and probabilities (c) at two values of $i_{THz}$. Increasing of $i_{THz}$ shifts the curves to lower currents. Since underdamped JJs switch to $V \simeq V_c$, the time-average voltage is determined by the switching probability,

$$\langle V(I)\rangle \simeq G(I)V_c.$$

Fig. S2 (d) shows simulated mean voltages as a function of bias current amplitude without (navy) and with a

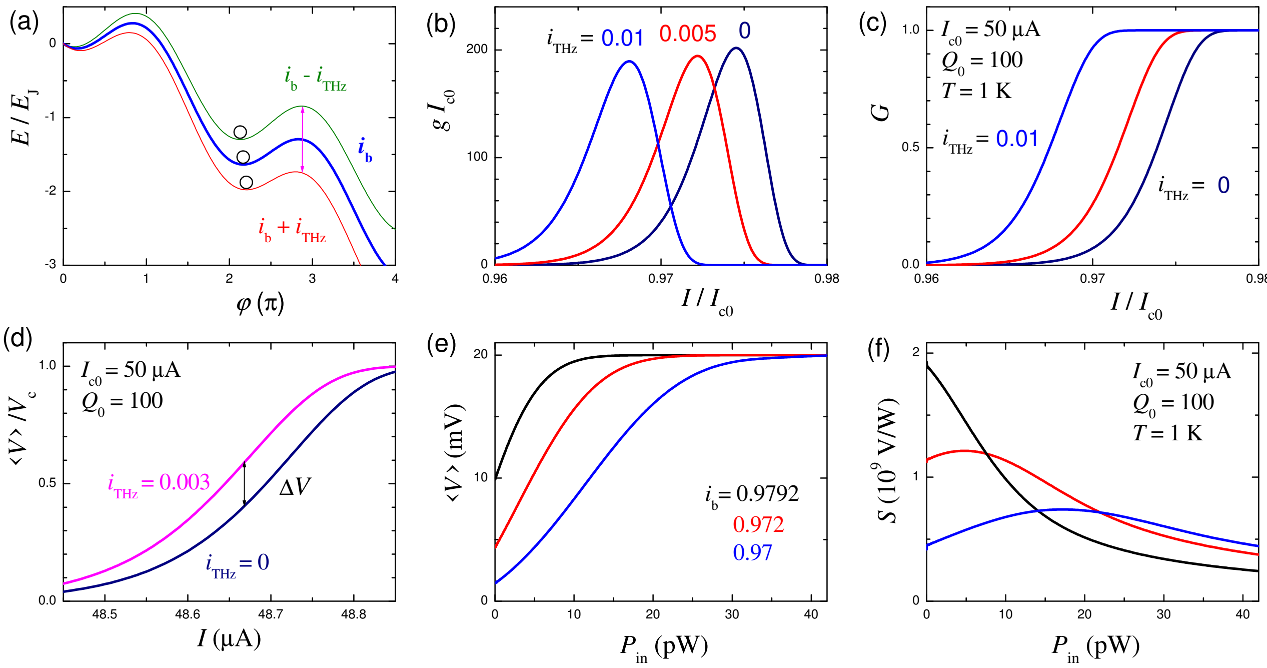


FIG. S2. **Operation of a single-junction SCD**$_1$. (a) Josephson wash-board potentials at a bias current, $I/I_{c0} = 0.5$ (blue). The EMW-induced current, $I_{THz}$, shakes the potential, as indicated by red and olive curves, increasing the escape rate. (b) Calculated switching current histograms (probability densities) at small EMW amplitudes $I_{THz}/I_{c0} = 0$ (navy), 0.005 (red) and 0.01 (blue). (c) Switching probabilities for the same $i_{THz}$ as in (b). (d) The time-average current-voltage characteristics of the junction without MW (navy) and with $i_{MW} = 0.003$ (magenta). The vertical shift of the curves, $\Delta V$, represents the SCD response at a given bias current. (e) Time-average voltage responses of a (non-resonant) SCD$_1$ as a function of incoming EMW power at three bias current amplitudes. (f) Detector sensitivities, $S = \Delta V/P_{in}$, for the curves from (e). Simulations are made for a single JJ with $I_{c0} = 50$ $\mu$A, $Q_0 = 100$, $T = 1$ K, and $R_{THz} = 100$ $\Omega$. Data from Ref. [2] with voltage scales in (e) and (f) scaled to $V_c = 20$ mV of Bi-2212 IJJ.

small $i_{THz} = 0.003$ (magenta) [2]. The voltage response, $\Delta V_1$, of a single JJ is equal to the vertical shift of the curves. At small $i_{THz}$, it is proportional to the slope and the shift of the step-like $G(I)$ [2].

$$\Delta V_1 \simeq -V_c \frac{\partial G}{\partial i_s} \frac{\partial i_s}{\partial i_{THz}} i_{THz}. \tag{S5}$$

The maximum sensitivity is achieved at the steepest part in the middle of the step, $G \simeq 0.5$, where the slope can be expressed via the width of the switching histogram, $\partial G/\partial i_s \simeq I_{c0}/\delta_{s1}$. Therefore, at optimal operation,

$$\Delta V_1 \simeq -V_c \frac{I_{c0}}{\delta_{s1}} \frac{\partial i_s}{\partial i_{THz}} i_{THz}. \tag{S6}$$

The sensitivity $S_1$(V/W) is defined as the ratio of the voltage response to the incoming power,

$$S = \frac{\Delta V}{P_{in}}.$$

To calculate it, we need to define the relation between $i_{THz}$ and $P_{in}$. To couple them, first we need to introduce a high-frequency impedance $Z_{THz}$ of the detector, so that $V_{THz} = Z_{THz} I_{THz}$. The absorbed MW power is then

$$P_a = \frac{I_{THz}^2 R_{THz}}{2}, \tag{S7}$$

where $R_{THz} = \mathrm{Re}(Z_{THz})$. $Z_{THz}$ represents the total (environmental) high-frequency impedance of the device, including electrodes, which should be designed and act as antennas for effective catching of EMW [5]. Generally, $R_{THz} \neq R_{QP}$.

Second, we need to introduce the absorption efficiency,

$$\chi = \frac{P_a}{P_{in}}.$$

The maximum value, $\chi = 0.5$, is achieved at the impedance matching condition [6]. However, in reality $\chi$ is somewhat lower because of the leakage QP current [5]. Thus,

$$P_{in} = \frac{I_{THz}^2 R_{THz}}{2\chi}. \tag{S8}$$

The values of $R_{THz}$ and $\chi$ are often ill-defined. In what follows we will use $R_{MW} = 100$ $\Omega$ [7] and $\chi = 0.5$, assuming the best case scenario of a well-matched and optimized detector [5, 6].

### Sensitivity of a single-junction SCD

Figs. S2 (e) and (f) shows numerically modeled voltage responses and sensitivities of a single junction at three bias current amplitudes. Calculations are made for a Bi-2212 IJJ with parameters: $I_{c0} = 50\ \mu$A, $V_c = 20$ mV, $Q_0 = 100$, $T = 1$ K. Fig. S2 (e) shows time-average voltage versus the incoming power, $P_{in}$, for three bias amplitudes, $i_b = I_b/I_{c0}$, close to the most probable switching current in the absence of radiation. Fig. S2 (f) shows corresponding sensitivities. The largest sensitivity at low $P_{in}$ is achieved for the black curve at bias current corresponding to the middle of the steps $G(i, i_{MW} = 0) \simeq 0.5$. With increasing $P_{in}$ the response saturates at $V = V_c$, the voltage response becomes independent of $P_{in}$ and the sensitivity decays as $1/P_{in}$. The saturation occurs when the shift of the histogram exceeds the full width of the histogram, $\sim \delta I_{s1}$, as for the blue curve at $i_{THz} = 0.01$ from Fig. S2 (b). By reducing the bias current below the optimal point it is possible to extend the dynamic range at the expense of lower sensitivity.

The THz sensitivity of a non-resonant single-junction SCD at $\omega > \omega_p$ can be estimated as [2],

$$S \sim 2\chi V_c g_{max} I_{c0} R_{THz} \frac{e^2}{(\hbar\omega)^2}. \qquad \text{(S9)}$$

where $g_{max}$ is the maximum switching probability density and $\omega$ is the EMW angular frequency. Taking into account that $g_{max}\delta I_{s1} \simeq 1$, Eq.(S9) can be rewritten as,

$$S \sim 2\chi V_c R_{THz} \frac{e^2}{(\hbar\omega)^2} \frac{I_{c0}}{\delta I_{s1}}. \qquad \text{(S10)}$$

Thus, the narrower is the switching histogram, the higher is the SCD sensitivity. Using Eq. (S4) and taking into account that $V_c = I_{c0} R_{QP}$, Eq. (S10) can be rewritten as,

$$S \sim \chi \frac{R_{THz}}{R_{QP}} \left(\frac{2E_{J0}}{k_B T}\right)^{2/3} \left(\frac{V_c}{V_\omega}\right)^2 I_{c0}^{-1}, \qquad \text{(S11)}$$

where $V_\omega = \hbar\omega/2e$ is the voltage of primary Shapiro step. For a Bi-2212 junction with $V_c = 20$ mV, $I_{c0} = 50\ \mu$A at the radiation frequency 1 THz, $T = 1$ K and $R_{THz} \sim R_{QP}$, one obtains, $S \sim 1 \times 10^9$ (V/W), consistent with the values in Fig. S2 (f).

## SIV. SWITCHING STATISTICS IN BI-2212

### Switching of a single intrinsic Josephson junction.

Switching statistics of conventional low-$T_c$ JJs is very well studied [3]. Switching statistics of a single IJJ has been also reported, e.g., in Refs. [8–10]. Fig. S3 represent the corresponding data for a single Bi-2212 IJJ. It is well described by the conventional thermal-activation escape, Eqs. (S1-S3), except close to $T_c$, where the collapse of thermal activation occurs due to enhanced retrapping associated with the reduction of $Q_0$ [8, 9].

From Fig. S3 (a) it can be seen that the switching histogram at 4.2K is narrow with $\delta I_{s1} \simeq 1\ \mu$A, less then 1 % of $I_{c0} \simeq 140\ \mu$A.

### Multi-junction switching

Figure S4 (a) shows the $I$-$V$ curve of the detector mesa (the same as in Fig. 5 of the manuscript) at MW irradiation. This measurement is made at a fixed bias amplitude. Nevertheless, the $I$-$V$ exhibits multiple branches, corresponding to different number of activated JJs. An underdamped JJ is bi-stable. Once switched, it stays in the resistive state until the current is reduced below the retrapping current. Arrays of underdamped JJs are multi-stable because different number of JJs can be in zero or resistive states. The number of activated JJs, $n$, at the raising part of the $I$-$V$ is affected by fluctuations. Upon ramping down the current, $n$ stays constant leading to recording of a specific $I$-$V$ branch. In the next cycle, $n$ may be different and another branch will be recorded. The $I$-$V$s in Figs. 5 (d) and 6 (e) were measured at $f_b = 23$ Hz during 1 s and contain 23 bias periods. Thus, the multi-branch structure seen in the $I$-$V$s is due to statistical variation of $n$ at different bias cycles.

From Fig. S4 (a) it is seen that IJJs switch sequentially in the extended current range 31 $\mu$A $< I <$ 38 $\mu$A, indicating some spread in critical currents. Figs. S4 (b) and (c) show a 3D and a color plot of switching current histograms at different voltage levels. It can be seen that the width of switching histograms at a given voltage is narrow, $\sim 1\ \mu$A. Constant voltage implies constant number of switched IJJs. Therefore, such statistics can be compared with the single-junction case. Thus, this mesa belongs to the class of cascade detectors in which $\delta I_{sn} \sim 5 - 6\ \mu$A is broader than $\delta I_{s1} \sim 1\ \mu$A.

## SV. LOCK-IN READOUT

Lock-in technique measures (usually the primary) Fourier component of an object excited by a harmonic current, $I = I_b e^{i\omega_b t}$. Ideally, the voltage response, $V(t)$, is periodic with the same period $\tau = 2\pi/\omega_b$. In this case the spectrum contains only harmonics of $\omega_b$, given by the Fourier series,

$$V_k = \frac{1}{\tau} \int_{-\tau/2}^{\tau/2} V(t) e^{-ik\omega_b t} dt, \quad (k - \text{integer}). \qquad \text{(S12)}$$

In reality, the response is never perfectly periodic due to noise and drift. Therefore, averaging over several periods is used to reduce the statistic noise.

Thus the lock-in readout yields the Fourier component at the excitation frequency, $V_f$, Eq. (S12), which for a harmonic $V(t)$ coincides with the voltage amplitude

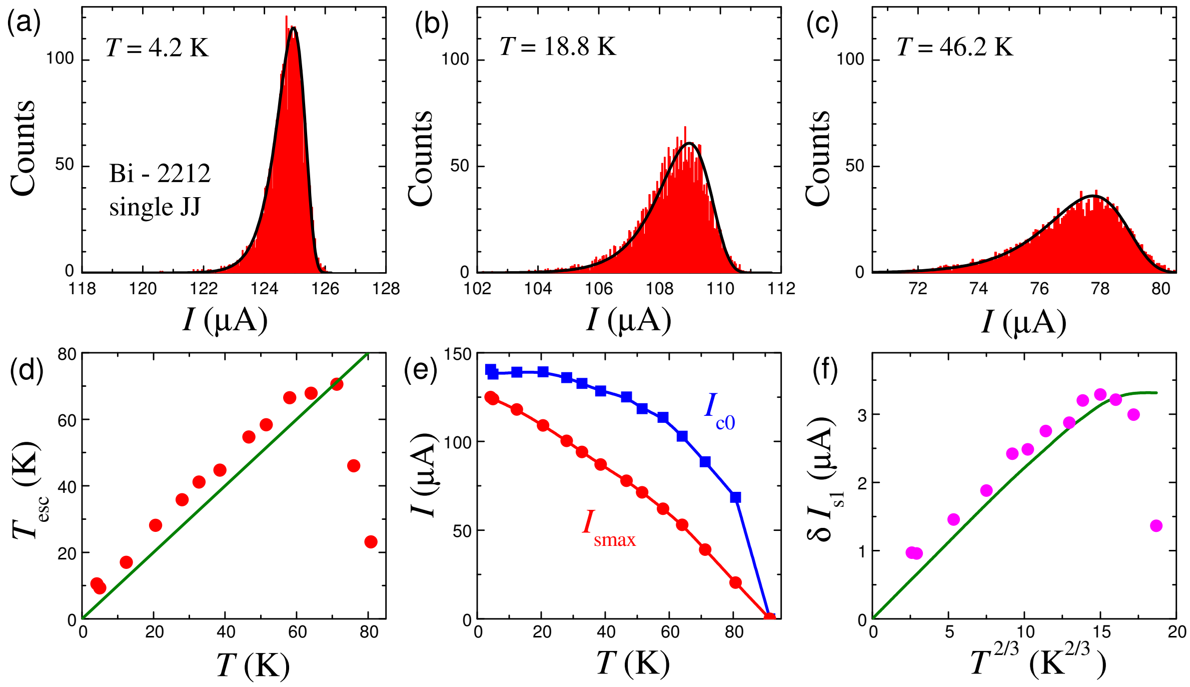


FIG. S3. **Switching statistics of a single Bi-2212 intrinsic Josephson junction without external radiation.**. (a)-(c) Switching current histograms at different temperatures. Red columns represent measured histograms, black lines are calculated switching probability densities, Eq. (S2), using $T_{esc}$ and $I_{c0}$ as fitting parameters. (d) The effective escape temperature, $T_{esc}$, versus bath temperature. The straight line indicates the expected $T_{esc} = T$ dependence. (e) Temperature dependencies of the measured most probable switching current, $I_{smax}$ (red circles), and the fluctuation-free critical current, $I_{c0}$ (blue squares), obtained from fitting. (f) Symbols represent measured full-width at half-maximum of switching histograms versus $T^{2/3}$. The solid line represents the expected dependence, Eq. (S4), for $I_{c0}(T)$ from (e). Data in (a-e) is from Refs. [8–10].

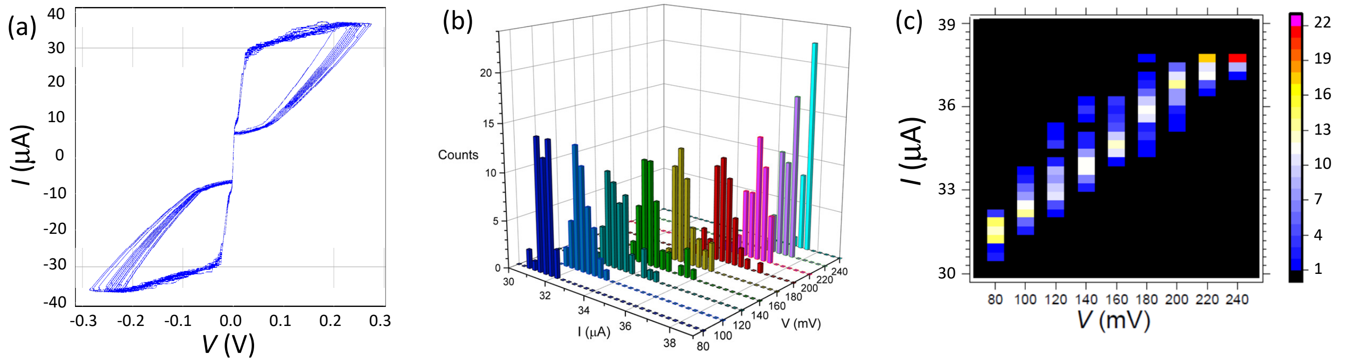


FIG. S4. **Multi-junction switching statistics in a Bi-2212 mesa.** (a) The *I*-*V* characteristics of the same Bi-2212 mesa as in Fig. 5 of the manuscript with applied MW radiation. (b) Switching current histograms at different voltage levels. (c) The color plot of histograms from (b).

(measured in Volts). However, the noise floor depends on the duration of measurement and is, therefore, expressed in units of $V/Hz^{1/2}$. The main advantage of the lock-in technique is that its noise floor can be much lower than for dc-measurements. This occurs because the Fourier integral has a narrow band-width and can effectively filter away noise contributions at other frequencies. On the other hand, the noise of the dc-measurements is given by integration of the whole noise spectrum (up to some setup-specific high-pass cut-off).

SCD, both single junction and cascade, has a background voltage $V(0) \neq 0$ at $P_{\rm THz} = 0$. For example, at the optimal bias for SCD$_1$, $G = 0.5$ and $\langle V_1(0)\rangle \simeq 0.5V_c$. For SCD$_n$ operating in the pure cascade gain mode,

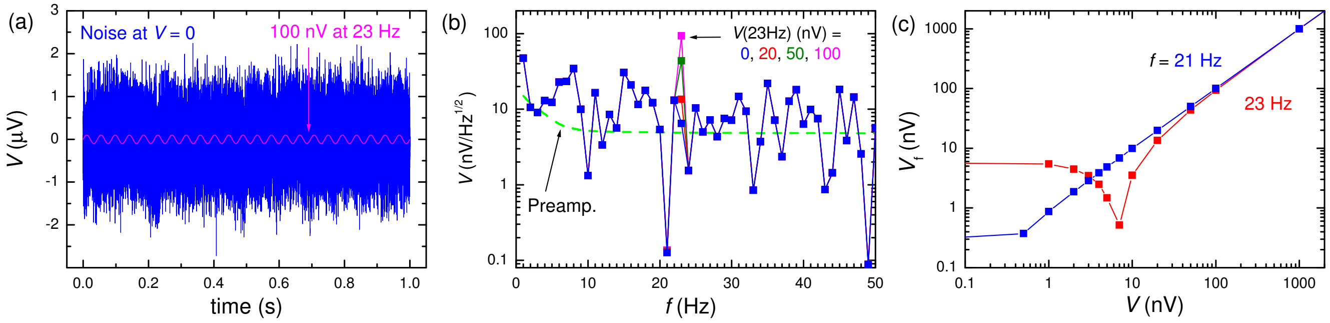


FIG. S5. **Preamplifier noise characteristics at zero voltage.** (a) The blue line represents a measured data sequence of 100000 samples over 1 s time interval. Here the bias current is applied, but the JJs stay in the superconducting state (do not switch). The magenta line shows a small harmonic signal superimposed on the data for the analysis of lock-in resolution. (b) Blue symbols represent the DFT spectrum of the noise data from (a). The green dashed line shows the intrinsic noise of the preamplifier, according to producer specification. Red, olive and magenta symbols show spectra with superimposed harmonic signal of 20, 50 and 100 nV at $f = 23$ Hz. (c) The lock-in signal, $V_f$, versus the signal amplitude at $f = 23$ Hz (red) and 21 Hz (blue). It is seen that the resolution $\sim$ nV (for 1s measurement time) is determined by the preamplifier noise floor.

$V_n(0) \simeq n_0 V_c$. The detector signal is given by the voltage difference $\Delta V(P) = V(P) - V(0)$.

## SVI. NOISE MEASUREMENTS

The SCD has two sources of noise: the external readout noise (in our case determined by the preamplifier) and the internal statistic uncertainty caused by the probabilistic switching of JJs.

### Preamplifier noise

Fig. S5 (a) represents preamplifier noise measurements in the zero-voltage state (with applied current at $f_b = 23$ Hz). There are in total 100000 points measured over $\tau =$ 1 s time interval with equal time intervals of $dt = 10$ $\mu$s.

Blue symbols in Fig. S5 (b) represent the Fourier spectrum of the data sequence from (a). The Fourier integrals, Eq. (S12), are calculated using the standard discrete Fourier transform (DFT) algorithm with a rectangular window. The noise floor $\sim 5$ nV/Hz$^{1/2}$ is consistent with the specification of our room-temperature preamplifier (AMP01), depicted by the green dashed line. It is much smaller than the apparent dc-noise $\sim 1$ $\mu$V in Fig. S5 (a).

To determine the lock-in resolution, we added a small harmonic signal to the noise data, as shown by the magenta line in Fig. S5 (a), and analyzed the threshold of its distinctiveness in the lock-in signal. Red, olive and magenta symbols in Fig. S5 (b) show Fourier spectra of noise plus signal with amplitudes 20 (red), 50 (olive) and 100 (magenta) nV at $f_b = 23$ Hz. It can be seen that the lock-in signal is clearly distinct at $f = f_b$ and merges with the noise (blue) at all other frequencies.

Fig. S5 (c) represents the lock-in response, $V_f$, versus the signal amplitude at $f_b = 23$ (red) and 21 (blue) Hz. For $f_b = 23$ Hz, the signal becomes resolved at $V > V_{min} \simeq 10$ nV, which is much smaller than the dc-noise floor. This illustrates the advantage of lock-in measurement.

### Statistical noise of the SCD

The statistic uncertainty is caused by the probabilistic switching of JJs. For a single JJ, the relative voltage uncertainty for 1 s measurement is given by Eq.(4) in the main text. The nominator in that equation represents the binomial (Bernoulli) variance for a single switching event and the denominator represents the statistical averaging of $2f_b$ switching events at positive and negative bias maxima during the 1 s time interval. At the optimal sensitivity, $G = 0.5$, and $f_b = 23$ Hz, the corresponding telegraph noise is rather large, $\delta V_1/V_c \sim 0.1$.

The relative voltage uncertainty of a cascade-SCD is given by Eq. (7) in the main text and depends both on the operation mode and array uniformity, $\delta n$. As explained in the main text, cascading may reduce the relative statistical uncertainty. For example, if we look at the histograms from Fig. S4 (c), the voltage spread at a fixed current is $\sim \pm 20$ mV, while the mean voltage is $\sim 200$ mV, yielding a factor 10 reduction of uncertainty. Thus, for this mesa the relative statistic uncertainty of voltage readout would be in the range $10^{-2}$.

Fig. S6 (a) shows a time sequence of voltage for a Bi-2212 mesa at $f_b = 23$ Hz and the MW attenuation of 3.7 dB (the black $I$-$V$ from Fig. 3 (d)). It is seen that the array always switch at positive and negative bias maxima, indicating that the switching probability $G_n^* =$ 1. As explained in the main text, this corresponds to the pure cascade gain operation mode. Unlike the single-junction SCD, shown in Fig. S1, here the signal is caused not by switching (or not switching) of the JJ, but by the

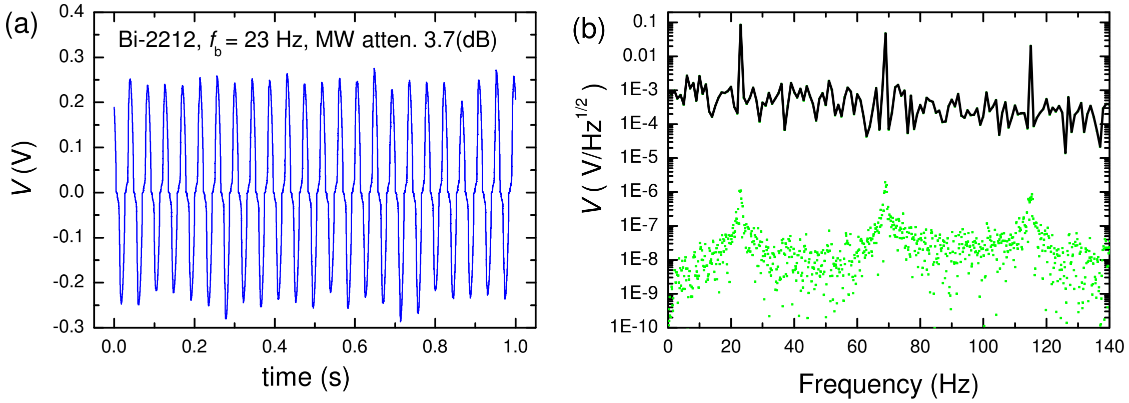


FIG. S6. **Lock-in response of Bi-2212 cascade SCD.** (a) Time dependence of voltage response for a Bi-2212 mesa over 1 s interval. The $V(t)$ corresponds to the black $I$-$V$ in Fig. 5 (d) for MW attenuation of 3.7 dB. Note that the array always switch at positive and negative bias maxima, indicating the pure cascade gain operation mode with $G_n^* = 1$. (b) Black lines represent the straightforward DFT spectrum of $V(t)$ from (a). Green dots represent the actual noise along the $I$-$V$. The disparity with the black line indicates that the noise floor is dominated by the statistical uncertainty.

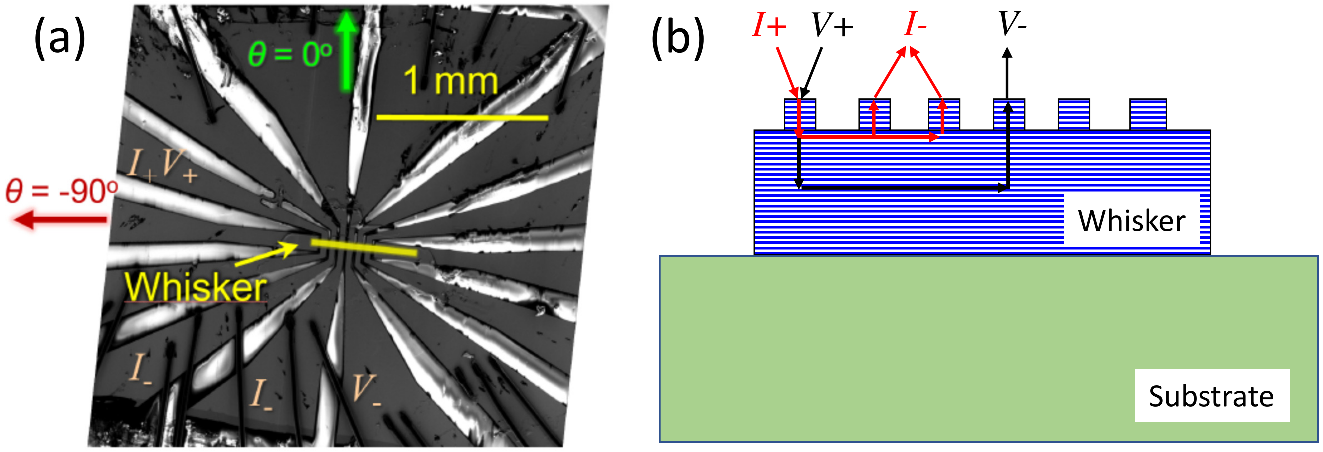


FIG. S7. **Geometry and bias schematics of the Bi-2212 detector**. (a) SEM image of the studied Bi-2212 device used for polarization loss diagram analysis. The green and the red arrows indicate polarization directions $\Theta = 0°$ and $\Theta = +90°$, respectively. The whisker is indicated by the yellow line at the center. Current and voltage contancts are marked. (b) A sketch of the bias schematics for four-probe measurements.

number of switched JJs. The compound (non-binomial) statistical uncertainty is reflected in the variation of the peak voltages.

The black line in Fig. S6 (b) represents the DFT spectrum of the waveform from (a). The detector signal is represented by the primary peak at $f = f_b = 23$ Hz and odd harmonics, which appear due to non-sinusoidal shape of $V(t)$ caused by non-linearity of the $I$-$V$ [11]. Remarkably, the signal floor, $\sim 2\ 10^{-4}$ (V/Hz$^{1/2}$) at 23 Hz, is much higher than the actual measurement noise along the $I$-$V$, $\sim 10^{-8}$ (V/Hz$^{1/2}$), as shown by green dots in Fig. S5 (b). This disparity indicates that the measurement is dominated by statistical uncertainty. However, the relative uncertainly, $\delta V/V_f \simeq 2\ 10^{-4}/0.1 = 2\ 10^{-3}$, is 50-times better than 0.1 expected for telegraph noise in a single JJ. This clearly demonstrates the partial obviation of telegraph noise by SCD$_n$.

## SVII. EXPERIMENTAL SETUP

### Bias configuration

SCD is based on a current-biased JJs. However, current sources usually have some (negative) load-line resistance, $R_l$ (the ideal current bias corresponds to $R_l = \infty$). We use a programmable function generator, with a fairly large $R_l \sim 100$ kOhm. Since $R_l$ is negative, switch-

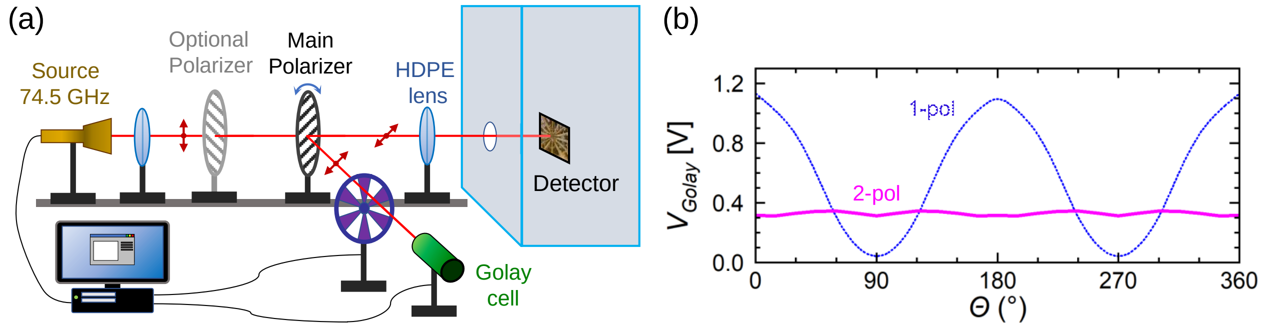


FIG. S8. (a) A sketch of the experimental setup for MW measurements. It includes a MW source, two HDPE lenses, one (1-pol) or two (2-pol) polarizers, a Golay cell detector and an optical cryostat in which the detector is kept at $T = 3.4$ K. (b) The Golay cell voltage, as a function of the polarization angle, $\Theta$, of the incoming MW beam. The blue and magenta lines represents measurements with one and two polarizers, correspondingly. It is evident in the plot that in the 1-pol configuration $P = P_0 \cos^2(\Theta)$, and in the 2-pol configuration, $P \simeq P_0/4$.

ing of additional JJs leads to a reduction of current by, $\Delta I = \Delta V/R_l$, which makes current sub-critical and allows observation of small fractions of different branches on the raising part of the $I$-$V$, as can be seen in Fig. 5 (b). The load-line effect is visible in Fig. 5 (d) for a Bi-2212 mesa. It can be seen that at large voltages, $V \sim 0.3$ V, the current decreases by $\Delta I = \Delta V/R_l \sim 3$ $\mu$A, yielding $R_l \sim 100$ kOhm. For Nb-arrays with small $V_c \simeq 0.2$ mV the load-line effect is negligible. Thus, we are dealing with a slightly non-ideal current bias. The finite load-line resistance has a minor effect on SCD operation. It only slightly distorts the $I(t)$ waveform, affecting the current ramp rate, which has a small (logarithmic, compared to the effect of $P_{THz}$) influence on the switching statistics.

All measurements are performed in a quasi-4-probe configuration. Fig. S7 represents (a) a mm-scale SEM image and (b) bias schematics of a Bi-2212 device. The bias current is sent through one of the bias electrodes, marked $I_+$ in Fig. S7 (a), to a small mesa, and is taken out through the whisker via two large mesas marked $I_-$. The large difference in areas and critical currents between $I_+$ and $I_-$ contacts ensures that only IJJs in the small mesa switch into the resistive state, forming the detector readout signal. All other mesas on the whisker remain in the zero-voltage state. The voltage at the mesa is measured through a pair of contacts, $V_+$ via a separate wire on the same electrode as $I_-$, and $V_-$ on a separate mesa. Due to a large anisotropy of Bi-2212, there is no voltage drop along the whisker. Therefore, such contact configuration allows accurate measurement of the voltage solely in the mesa.

### Microwave measurements

Supplementary Fig. S8 (a) represents a sketch of the experimental setup. Measurements are performed in a closed-cycle optical cryostat at zero (ambient) magnetic field. The cryostat is cooled by a pulse-tube cryocooler down to the base temperature of $3.2-3.4$ K. We use a linearly polarized MW source operating at a frequency of 74.5 GHz. The MW beam is guided to the sample in a quasi-optical manner through an optical window of a cryostat with the help of two high density polyethylene (HDPE) lenses. The MW beam is focused on the front side of the sample along the normal to the substrate. We utilize either one or two grid polarizers positioned between the HDPE lenses to regulate the incoming MW power and polarization. The MW power is monitored by a Golay cell detector. To validate the setup, both configurations with one and two polarizers were tested by placing the Golay cell approximately at the sample position. Subsequently, during the measurements, we simultaneously measured the Golay cell voltage induced by a scattered (diffracted) beam from the polarizer. Both types of power monitoring yield consistent results.

In the case of a single polarizer (1-pol configuration), the rotation of the polarizer changes both the power and the polarization of the incoming beam. The blue line in Fig. S8 (b) shows the Golay cell voltage, $V_G$, as a function of the polarizer angle $\Theta$ in the 1-pol configuration. It closely follows the dependence, $V_G \propto \cos^2(\Theta)$, expected for the MW power. This observation shows that the Golay cell voltage is directly proportional to the MW power, $V_G \propto P_{MW}$.

In the two polarizer (2-pol) configuration, the incoming polarization is determined by the angle, $\Theta$, of the rightmost (close to the sample) polarizer. The angle of the leftmost (close to the source) polarizer, $\theta$, affects only the incoming power,

$$P(\theta, \Theta) = P_0 \cos^2\theta \cos^2(\Theta - \theta) \qquad \text{(S13)}$$

Here $P_0$ is the maximum power achieved when both polarizers are aligned with the polarization of the source: $\Theta = \theta = 0$. Since for grid polarizers states $\Theta$

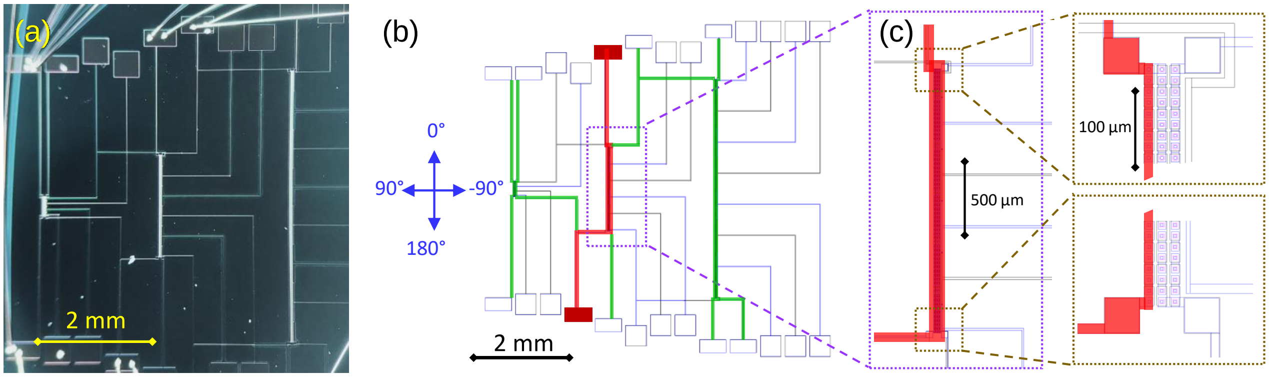


FIG. S9. **Geometry of the Nb-array.** (a) Optical image of the Niobium-based sample. The thick bright line in the center contains three parallel linear arrays of 128 JJs in series. We study one of them. (b) Schematics of Nb-based sample. We highlighted in red parts where the current flowed, in green other parts of the circuit electrically connected to the linear array we studied. On the left, in blue we show the system of reference for the MW polarization, Θ. (c) Zoom-in of the schematics over the area JJ array. The small squares outlined in magenta over the linear array represent the JJs.

and $\Theta \pm 180^\circ$ are equivalent, we should consider only two quadrants, namely $-90^\circ \leq \Theta \leq 90^\circ$. For a given $\Theta$, $P(\theta)$ reaches maximum at $\theta = \Theta/2$ with the lowest value, $P = P_0/4$, occurring at $\Theta = \pm\,90^\circ$. Therefore, it is possible to keep constant $P(\Theta) = P_0/4$ in the whole range of angles, $-90^\circ \leq \Theta \leq 90^\circ$, by adjusting the angle $\theta$ according the equation,

$$\cos^2\theta\cos^2(\Theta-\theta) = 1/4. \tag{S14}$$

The magenta line in Fig. S8 (b) shows measured $V_G$ versus $\Theta$, obtained in the 2-pol configuration with the angle $\theta$ of the leftmost polarizer adjusted according to Eq. (S14). We observe that the MW power stays practically constant at a quarter of the maximum in the 1-pol configuration. Thus, the main advantage of the 2-pol configuration is that it allows the rotation of polarization without changing the MW power. However, the 2-pol scheme with perfectly parallel polarizers is prone to formation of standing-wave resonances, which may strongly affect the transmission power. In order to prevent such resonances we place the polarizers as far as possible from each other and slightly tilt the leftmost polarizer along the vertical axis.

## SVIII. LINEAR NB/NB$_x$SI$_{1-x}$/NB ARRAYS

Figure S9 (a) represents a full-scale optical image of the studied Nb-based sample. It contains several arrays. We studied one of the 1.8 mm-long linear array containing 128 JJs, located in the center of the chip. The junctions are almost identical. Small observable variations of the switching current can be (at least partly) attributed to an ambient magnetic field.

The sample was fabricated at PTB. First the bottom part of the Nb electrodes was sputtered on the silicon substrate: it consists of 63 segments disposed along a line as well as the electrodes connecting the arrays to the pads. Then two $6 \times 6$ μm squares, consisting of a thin layer ( $30-40$ nm) of $\mathrm{Nb_xSi_{1-x}}$, where x $\simeq$ 0.14, are deposited: one on each edge of the electrode segment. These squares are the central layer of the junctions of the array. A layer of $SiO_2$ around the junctions is deposited to insulate the bottom electrodes. Finally the upper part of the electrode is deposited and it consists of 64 segments, which connect each two junctions in order to electrically connect all the components of the array.

In Figs. S9 (b) and (c) we show the biasing configuration: the electrodes where the current flowed are highlighted in red, while those not in use but still electrically connected to the linear array are marked in green. In panel (b) we reported in blue the frame of reference for the orientation of the sample refereed to the MW polarization Θ.

### Microwave response of the Nb-array in the one-polarizer configuration

In Fig. S10 (a) we show results of MW measurements performed on the Nb-array using the 1-pol configuration. Here blue symbols and the solid red line represent the lock-in resistance of the array, $R$, and the number of active junctions, $n$, respectively, as a function of the polarizer angle, Θ. The corresponding voltage response $V = RI_b$ is obtained using the bias amplitude $I_b = 3.1$ mA. The dashed magenta line representing the simultaneously measured Golay cell voltage, $V_G \propto P$. Fig. S10 (b) shows the corresponding angular diagram of the array responsivity, $\Delta R/V_G$.

$R(\Theta)$ and $n(\Theta)$ exhibit similar behavior until all JJs switch into the resistive state, leading to saturation of the cascade multiplication factor $n(\Theta) = N = 128$. Beyond this point, the resistance continues to increase, albeit at a much slower rate. This happens because the switching current continues to decrease with increasing power, as

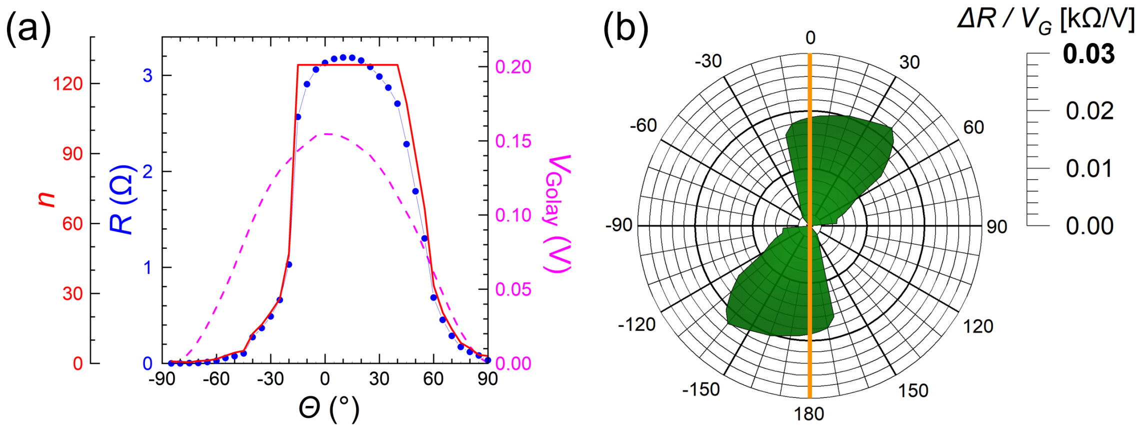


FIG. S10. **Microwave measurement of a Nb-array with the 1-pol configuration at $T = 3.5$ K.** (a) Lock-in resistance (blue symbols) and the number of active junctions, $n$, (red solid line) as a function of the polarization angle $\Theta$. The dashed magenta line represents simultaneously measured Golay cell voltage, proportional to the incoming MW power, $P \propto \cos^2(\Theta)$. (b) Polarization diagram for the sensitivity expressed as $\Delta R/V_G$. The corresponding voltage response $V = RI_b$ can be obtained using the bias amplitude $I_b = 3.1$ mA.

can be seen from the $I$-$V$s in Fig. 5 (d), obtained at high MW power. The reduction of $I_s(P)$ leads to an ordinary SCD response, without cascade amplification, which is apparently much weaker than for cascade-SCD.

Although, the overall results of the 1-pol measurements in Fig. S10 are similar to 2-pol measurements reported in Fig. 5 (f) of the manuscript, there are certain differences, which highlight the difference between 1-pol and 2-pol schemes. The key difference is caused by the varying MW power in the 1-pol case, compared to constant $P(\Theta)$ in the 2-pol case, as shown in Fig. S8 (b). Thus, the 2-pol scheme probes solely antenna effects associated with the sample geometry. The response in the 1-pol case is much more complicated due to the strongly non-linear $R(P)$ sensitivity. Furthermore a factor four larger maximum power in the 1-pol case leads to an extended saturated range, $n = 128$, which results in a quasi-flat top of the lobe in Fig. S10 (b), as compared to a much sharper tip in Fig. 5 (f).

## SIX. BI-2212 MESA STRUCTURES

Fig. S7 (a) shows a large-scale image of the studied Bi-2212 device. It is based on a whisker - a needle-like single crystal with typical sizes $(300-500) \times (20-30) \times (1-5)$ µm$^3$ along crystallographic $a$, $b$, and $c$ axes, respectively. Atomic scale IJJs are naturally formed along the $c$-axis. The fabrication process is the following. The whisker is glued to a sapphire substrate, cleaved for obtaining a fresh surface and rapidly covered by a protective gold layer. Electrode pattern is formed by photolithography and cryogenic reactive ion etching (CRIE), which preserves oxygen content in Bi-2212 during etching. Mesa structures, containing hundreds of stacked JJs, were made by CRIE. Mesas are formed in a self-alignment way at the overlap between electrodes and the whisker. Subsequently the sample is transferred in a dual-beam FIB and some mesas are trimmed and reshaped to reduce the size. Finally, the sample was glued to a PCB, and bonding wires are attached to perform electric measurements.

### Microwave measurements of Bi-2212 samples using one-polarizer configuration

Fig. S11 represents MW measurements on the Bi-2212 device using the 1-pol configuration. Panels (a), (b) and (c) show resistances (blue symbols) and the number of active junctions (red solid lines in (b) and (c)) as a function of the polarization angle $\Theta$, obtained at three bias current amplitudes, $I_b = 8$, 25.3 and 38.1 $\mu$A. The dashed line in (a) shows the Golay cell voltage, which is the same for all experiments presented in this Figure.

At small bias amplitude, Fig. S11 (a), only one, $n = 1$, weakest surface junction is active during the full measurement. The top junction of a mesa exhibits a significantly smaller critical current, $I_s(1)$, reduced by a factor of $2-5$ in comparison to the majority of the other junctions, due to a short exposure to air after cleaving. Therefore, Fig. S11 (a) represents a conventional single-junction SCD response.

At higher bias, avalanche switching of multiple junctions occur in the bulk of the mesa. The corresponding cascade amplification of the readout voltage leads to the increase of the resistive response. For the case of a single JJ (a), the resistance varies between $\sim 280\ \Omega$ (the value with turned-off MW) to $\sim 320\ \Omega$. Thus the maximum response is $\Delta R \simeq 40\ \Omega$ is about 15% of $R$ in the case the MW beam is off. At the intermediate bias (b) the resistance ranges from 800 $\Omega$ up to about 4 k$\Omega$: an increase

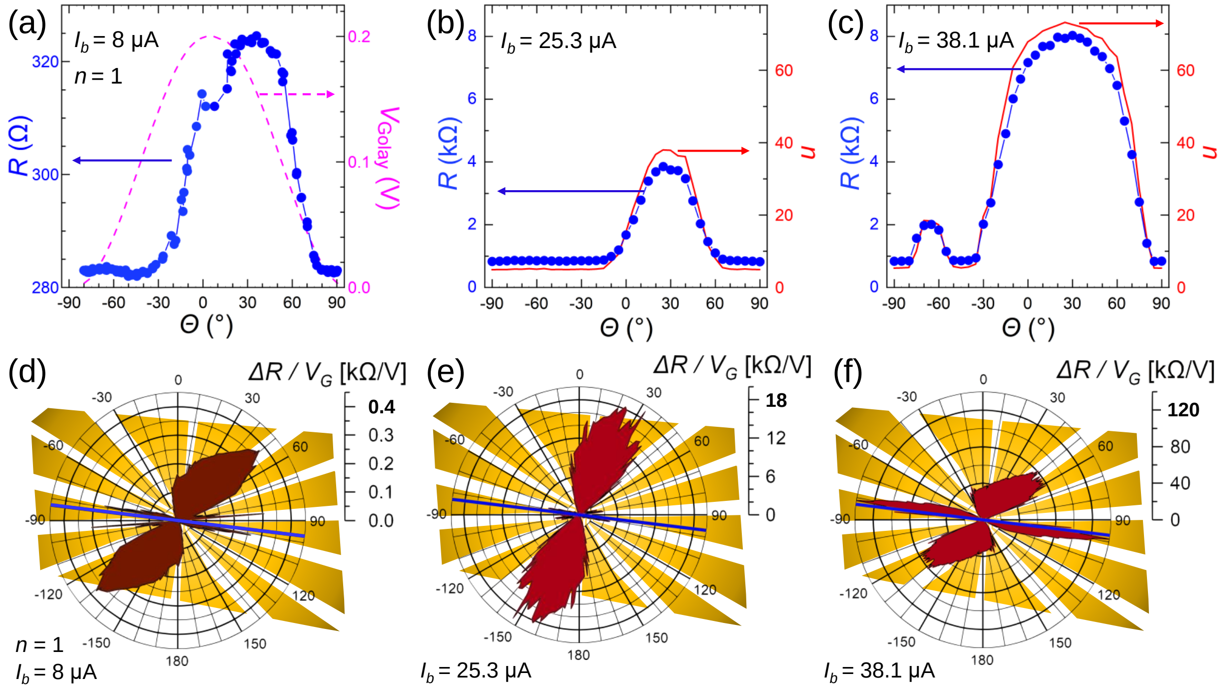


FIG. S11. **Microwave measurements on the Bi-2212 device in the 1-pol configuration.** (a-c) Angular dependencies of resistances (blue symbols) and (d-f) Polarization loss factor diagrams obtained on the same mesa using different bias amplitudes (a,d) $I_b = 8$ mA, (b,e) $I_b = 25.3$ mA, and (c,f) $I_b = 38.1$ mA at $T = 3.4$ $K$. A large difference in responsivities, cf. scales in (d-f), is associated with the variation of the cascade gain from $n = 1$ in (a) to 40 in (b) and about 70 in (c) as shown by solid red lines in (b) and (c). The dashed magenta line in (a) shows angular dependence of the Golay cell voltage (the same for all measurements). The blue line in (d-f) indicates the orientation of the whisker and the yellow background shows the large-scale electrode geometry. Voltage responses $V = RI_b$ can be obtained using the actual bias amplitudes.

of almost 400 %. At the highest bias, $I_b = 38.1$ μA, (c), $R$ increases from $\sim 800$ Ω up to $\sim 8$ kΩ, an increase of almost 1000 %. Voltage responses $\Delta V = \Delta R I_b$ can be obtained using the actual bias amplitudes. For a single IJJ case (a) $\Delta V = 0.32$ mV, for the sub-optimal cascade SCD (b) $\Delta V \simeq 81$ mV, and for the optimal case (c) $\Delta V \simeq 274.3$ mV. Thus, the voltage response increases almost by three orders of magnitude.

Such increase of responsivity is associated with the maximum cascade gain, $n$, which increases from 1 in (a) to approximately 40 in (b) and more than 70 in (c). A small increases in $I_b$ up to a value just below the $I_s$ of the majority of IJJs of a mesa, produces a dramatic increase in sensitivity of the detector, leveraging the amplification due to a massive amount of junctions switching together from a superconducting to an active (or resistive) state. In our devices such value is $\sim$ 40 μA, demonstrating that the advantage of such technique lies in the cascade amplification of the readout voltage.

Figs. S11 (d-f) show polarization loss factor diagrams, $\Delta R/V_{\mathrm{G}}$, obtained from the data in panels (a-c). We observe that the maximum sensitivity $\Delta R/V_G$, increases from 0.4 kΩ/V in (d), to $\sim 18$ kΩ/V in (e) and $\sim 100$ kΩ/V in (f).

As visible from Fig. S7 (a), the electrode sizes are suitable for the coupling with our MW source ($\lambda_0 \simeq 4$ mm). Furthermore, we observe that the directions of the two I$_-$ and the V$_-$ contacts are approximately aligned with the larger lobes observed in Fig. 5 (f). The observed four-fold polarization-loss diagram indicates that both the whisker and the top gold electrodes are participating in absorption of radiation. This confirms our conclusion that Bi-2212 whisker-based devices enable good impedance matching with free space due to the turnstile antenna geometry.

As in case of the Nb-array, we observe that 1-pol and 2-pol measurement schemes yield qualitatively similar but quantitatively different results, which is caused by the non-linearity of response combined with the large variation of power in the 1-pol case. Therefore, the simplicity of the 1-pol case is negated by the complexity of interpretation. Therefore, in the main text we show only 2-pol measurements, which reflect solely antenna effects associated with the geometry of the devices.

## SX. SELF-HEATING IN THZ GENERATION-DETECTION EXPERIMENT

Josephson junctions are prone to self-heating at high bias. Self-heating is one of the main limitations for THz emitters based on Bi-2212 mesas [12–19]. The to-

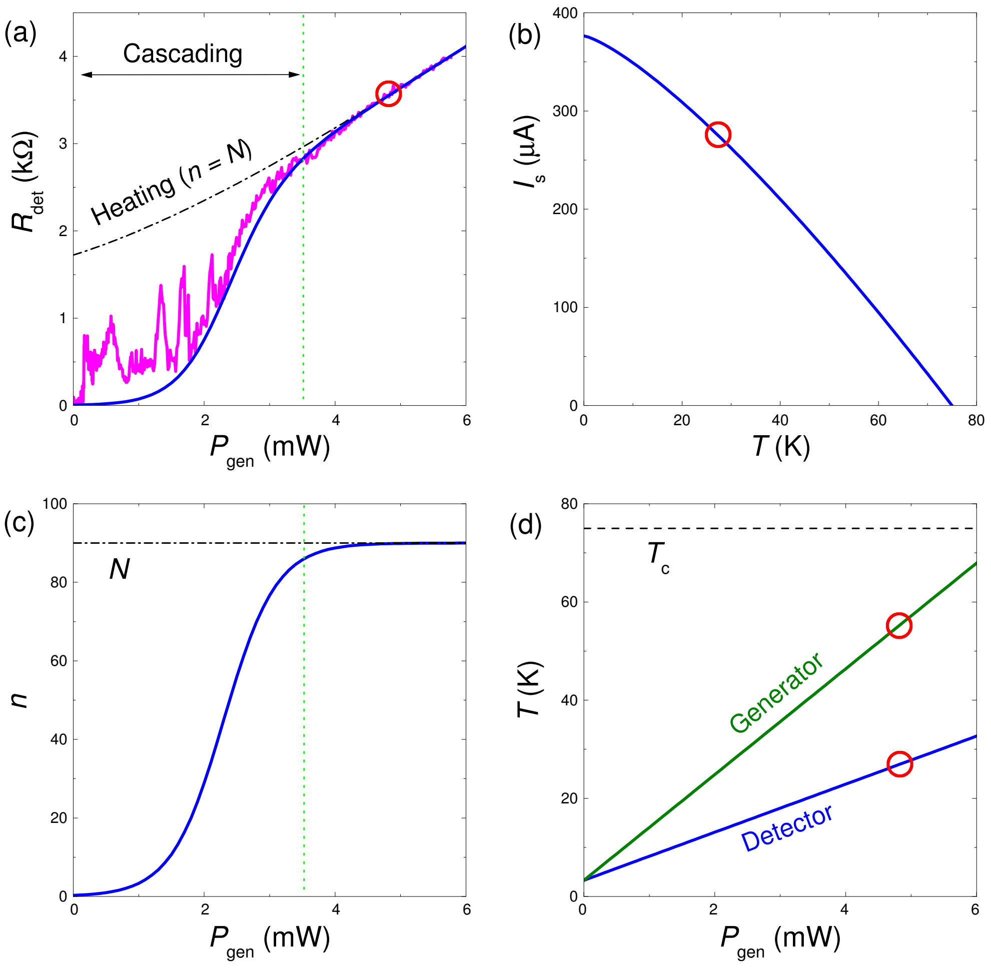


FIG. S12. **Estimation of self-heating drift of Bi-2212 detector response.** (a) The magenta line shows the THz generation-detection data from Fig. 4 (a) in a broader bias range. The black dashed-dotted line is the calculated upper limit of heat-induced drift assuming that all $N = 90$ IJJs are in the resistive state. The solid blue line is the heat-induced background for the number of active junctions, shown in (c). The difference between magenta and blue lines represents the THz radiation signal, which is dominated by the cascade amplification. (b) Temperature dependence of the mean switching current in the absence of radiation. (c) The fit of the number of active junctions as a function of heating power in the absence of radiation. (d) Estimated temperatures of generator and detector mesas, obtained from the experimental suppression of $I_s$ in the red $I$-$V$ from Fig. 4 (e), which is marked by red circles in (a), (b) and (d).

tal power dissipation in the generator, $P_{gen} = I_{gen}V_{gen}$, leads to the local temperature rise,

$$\Delta T_{gen} = P_{gen}R_{therm}, \tag{S15}$$

where $R_{therm}$ is the effective thermal resistance of the mesa. $R_{therm}$ depends on mesa size and geometry, [20], phonon mean-free path [21, 22], as well as on material parameters [23]. The THz emission vanishes when the local mesa temperature approaches the critical temperature, which for our whiskers is $T_c \simeq 75$ K. Since Josephson frequency is proportional to dc-voltage, self-heating limits the achievable upper frequency [12, 13].

In the THz generation-detection experiment from Figure 4 (d), not only the generator, but also a detector mesa was affected by heating of the near-by generator mesa. This leads to an upward drift of $R_{det}$. The magenta curve in Figure S12 (a) shows the same measurement in a broader bias range. It is seen that at $P_{gen} > 3.5$ mW, the emission peaks disappear and the detector response becomes a monotonous function of $P_{gen}$. As follows from Eq. (S15), the observed quasi-linear dependence is a signature of heating. Red circles in Fig. S12 correspond to the red $I$-$V$ in Fig. 4 (e) with $P_{gen} = 4.83$ mW. The mean switching current for this $I$-$V$, $I_s \simeq 277$ $\mu$A, is reduced in comparison with $I$-$V$s at low $P_{gen}$ with $I_s \simeq 370$ $\mu$A.

The reduction of $I_s$ is the consequence of temperature dependence of the switching current in the absence of radiation, as shown in Fig. S12 (b), consistent with earlier reports, see e.g. Fig. 4 in Ref. [10]. By matching the $I_s$ reduction we obtain the effective detector temperature

of $T_{det} \simeq 27$ K, as marked by the red circle in Fig. S12 (b). It is significantly larger than the base temperature of 3.3 K. The temperature at the generator is larger and decays with distance as $T(r) \simeq (r/a)T(0)$, where $a$ is the mesa size, see Fig. 4c in Ref. [22]. In our case the generator mesa size is $a = 10$ $\mu$m, the distance to the detector $r_{det} = 22$ $\mu$m, which leads to $T_{gen} \simeq 2.2 T_{det}$. Fig. S12 (d) shows thus obtained temperatures of generator and detector mesas as a function of $P_{gen}$. The corresponding thermal resistance of the generator mesa, $R_{therm} \simeq 10.8$ K/mW is consistent with anticipated values for similar size mesas [21, 23].

The knowledge of actual detector temperature allows estimation of heat-induced drift. The increase of resistance is caused by the reduction of $I_s(T)$. In the resistively shunted junction (RSJ) model, the lock in resistance is [11],

$$R = R_n \left[ 1 - \frac{1}{2}\left(\frac{I_s}{I_b}\right)^2 - \frac{1}{2}\left(\frac{I_r}{I_b}\right)^2 \right]^{\beta}. \tag{S16}$$

Here $I_r$ is the retrapping current, $I_b$ is the bias current amplitude, $R_n$ is the normal resistance and $\beta$ is a fitting parameter, which takes into account the non-linear shape of the $I$-$V$ curves. In the RSJ model with constant, bias-independent $R_n$, $\beta = 1$ [11].

The dashed-dotted line in Fig. S12 (a) shows calculated $R_{det}$ versus $P_{gen}$, using $T_{det}(P_{gen})$ and $I_s(T)$ from Figs. S12 (b) and (d) and the fitting parameter $\beta = 2$ reflecting bias dependent quasiparticle resistance in Bi-2212 IJJs. The retrapping current is small in this range, therefore, the 2nd term in Eq. (S16) is negligible. The value of $R_n \simeq 7$ k$\Omega$ corresponds to the resistance of the whole mesa with all $N = 90$ IJJs in the resistive state. Therefore, the dashed-dotted line represents the upper limit of heat-induced detector resistance.

The bias amplitude of the detector mesa has been chosen such that only few IJJs are active when $I_{gen} = 0$, see Fig. 4(e). However, the reduction of $I_s$ with increasing $P_{gen}$ leads to a gradual increase of the number of active junctions even without radiation. The corresponding fit $n(P_{gen})$ is shown in Fig. S12 (c). The solid blue line in Fig. S12 (a) represents the dashed-dotted line normalized by the number of active junctions, shown in Fig. S12 (c), $R_{det}(n) = (n/N) R_{det}(n = N)$. It provides a good estimation of the self-heating induced detector background in the absence of radiation.


[1] A. Barone and G. Paterno, Physics and Applications of the Josephson Effect (John Wiley & Sons, 1982),

[2] V. M. Krasnov. Resonant switching current detector based on underdamped Josephson junctions, *Phys. Rev. Appl.* **22**, 024015 (2024); Erratum, *ibid.* **23**, 049901 (2025).

[3] J. M. Martinis, M. H. Devoret, and J. Clarke, Experimental tests for the quantum behavior of a macroscopic degree of freedom: The phase difference across a Josephson junction. *Phys. Rev. B* **35**, 4682 (1987).

[4] L D. Jackel, W. W. Webb, J. E. Lukens, and S. S. Pei. Measurement of the probability distribution of thermally excited fluxoid quantum transitions in a superconducting ring closed by a Josephson junction. *Phys. Rev. B* **9**, 115 (1974).

[5] V. M. Krasnov, A distributed active patch antenna model of a Josephson oscillator. *Beilstein J. Nanotechnol.* **14**, 151-164 (2023).

[6] C.A. Balanis, Antenna Theory: Analysis and Design, 3rd ed.; John Wiley & Sons, Inc., Publ.: Hoboken, New Jersey, 2005.

[7] I. Siddiqi, R. Vijay, F. Pierre, C. M. Wilson, L. Frunzio, M. Metcalfe, C. Rigetti, R. J. Schoelkopf, M. H. Devoret, D. Vion and D. Esteve. Direct Observation of Dynamical Bifurcation between Two Driven Oscillation States of a Josephson Junction. *Phys. Rev. Lett.* **94**, 027005 (2005).

[8] V. M. Krasnov, T. Golod, T. Bauch and P. Delsing, Anticorrelation between temperature and fluctuations of the switching current in moderately damped Josephson junctions. *Phys. Rev. B* **76**, 224517 (2007).

[9] V. M. Krasnov, T. Bauch, S. Intiso, E. Hürfeld, T. Akazaki, H. Takayanagi, and P. Delsing, Collapse of Thermal Activation in Moderately Damped Josephson Junctions. *Phys. Rev. Lett.* **95**, 157002 (2005).

[10] V. M. Krasnov, T. Bauch, and P. Delsing, Probing the intrinsic Josephson coupling potential in $Bi_2Sr_2CaCu_2O_{8+\delta}$ superconductors by thermal activation. *Phys. Rev. B* **72**, 012512 (2005).

[11] R. A. Hovhannisyan, O. M. Kapran, T. Golod, and V. M. Krasnov, Accurate Determination of the Josephson Critical Current by Lock-In Measurements. *Nanomaterials* **11**, 2058 (2021).

[12] E. A. Borodianskyi and V. M. Krasnov, Josephson emission with frequency span 1-11 THz from small $Bi_2Sr_2CaCu_2O_{8+\delta}$ mesa structures, *Nat. Commun.* **8**, 1742 (2017).

[13] R. Cattaneo, E. A. Borodianskyi, A. A. Kalenyuk, and V. M. Krasnov, Superconducting Terahertz Sources with 12% Power Efficiency. *Phys. Rev. Appl.* **16**, L061001 (2021).

[14] L. Ozyuzer, A. E. Koshelev, C. Kurter, N. Gopalsami, Q. Li, M. Tachiki, K. Kadowaki, T. Yamamoto, H. Minami, H. Yamaguchi, T. Tachiki, K. E. Gray, W.-K. Kwok, and U. Welp, Emission of Coherent THz Radiation from Superconductors. *Science* **318**, 1291 (2007).

[15] T. M. Benseman, K. E. Gray, A. E. Koshelev, W.-K. Kwok, U. Welp, H. Minami, K. Kadowaki, and T. Yamamoto, Powerful terahertz emission from $Bi_2Sr_2CaCu_2O_{8+\delta}$ mesa arrays. *Appl. Phys. Lett.* **103**, 022602 (2013).

[16] T. Kashiwagi, T. Yamamoto, H. Minami, M. Tsujimoto, R. Yoshizaki, K. Delfanazari, T. Kitamura, C. Watanabe, K. Nakade, T. Yasui, K. Asanuma, Y. Saiwai, Y. Shibano, T. Enomoto, H. Kubo, K. Sakamoto, T.

Katsuragawa, B. Marković, J. Mirković, R. A. Klemm, and K. Kadowaki, Efficient Fabrication of Intrinsic-Josephson-Junction Terahertz Oscillators with Greatly Reduced Self-Heating Effects. *Phys. Rev. Appl.* **4**, 054018 (2015).

[17] H. Zhang, R. Wieland, W. Chen, O. Kizilaslan, S. Ishida, C. Han, W. Tian, Z. Xu, Z. Qi, T. Qing, Y. Lv, X. Zhou, N. Kinev, A. B. Ermakov, E. Dorsch, M. Ziegele, D. Koelle, H. Eisaki, Y. Yoshida, V. P. Koshelets, R. Kleiner, H. Wang, and P. Wu, Resonant Cavity Modes in $Bi_2Sr_2CaCu_2O_{8+x}$ Intrinsic Josephson Junction Stacks, *Phys. Rev. Appl.* **11**, 044004 (2019).

[18] Y. Ono, H. Minami, G. Kuwano, T. Kashiwagi, M. Tsujimoto, K. Kadowaki, R. A. Klemm. Superconducting Emitter Powered at 1.5 Terahertz by an External Resonator. *Phys. Rev. Appl.* **13**, 064026 (2020).

[19] M. Miyamoto, R. Kobayashi, G. Kuwano, M. Tsujimoto, and I. Kakeya, Wide-band frequency modulation of a terahertz intrinsic Josephson junction emitter of a cuprate superconductor. *Nat. Photon.* **18**, 267 (2024).

[20] Krasnov, V. M., Yurgens, A., Winkler, D., and Delsing, P. Self-heating in small mesa structures. *J. Appl. Phys.* **89**, 5578 (2001).

[21] Krasnov, V. M., Sandberg, M. and Zogaj, I. In situ Measurement of Self-Heating in Intrinsic Tunneling Spectroscopy. *Phys. Rev. Lett.* **94**, 077003 (2005).

[22] Krasnov, V. M., Katterwe, S. O. and Rydh, A. Signatures of the electronic nature of pairing in high-Tc superconductors obtained by non-equilibrium boson spectroscopy. *Nat. Commun.* **4**, 2970 (2013).

[23] M. M. Krasnov, N. D. Novikova, R. Cattaneo, A. A. Kalenyuk and V. M. Krasnov, Design aspects of $Bi_2Sr_2CaCu_2O_{8+\delta}$ THz sources: optimization of thermal and radiative properties. *Beilstein J. Nanotechnol.* **12**, 1392 (2021).
